# Threshold Effect of Environmental Regulation and Green Innovation Efficiency: From the Perspective of Chinese Fiscal Decentralization and Environmental Protection Inputs

**DOI:** 10.3390/ijerph20053905

**Published:** 2023-02-22

**Authors:** Liang Liu, Yuting Zhao, Xiujuan Gong, Shu Liu, Mengyue Li, Yirui Yang, Pan Jiang

**Affiliations:** 1 School of Economics and Management, Southwest University of Science and Technology, Mianyang 621010, China; 2 School of Environment and Resource, Southwest University of Science and Technology, Mianyang 621010, China

**Keywords:** environmental regulation, green innovation efficiency, environmental protection input, fiscal decentralization, threshold effect, DEA-SBM

## Abstract

In the context of China’s 14th Five-Year Plan and 2035 visionary goals of national economic and social development, to achieve the national dual carbon goals, an innovation-driven green development strategy must be implemented, and the relationship between environmental regulation and green innovation efficiency must be clarified. Based on the DEA-SBM model, in this study, we measured the green innovation efficiency of 30 provinces and cities in China from 2011 to 2020 by introducing environmental regulation as the core explanatory variable, and two threshold variables, environmental protection input and fiscal decentralization, to empirically analyze the threshold effect of environmental regulation on green innovation efficiency. We found that: (1) The green innovation efficiency of 30 provinces and municipalities in China is spatially distributed as strong in the east and weak in the west. (2) A double-threshold effect exists with environmental protection input as the threshold variable. Environmental regulation showed an inverted N-shaped relationship of first inhibiting, then promoting, and finally inhibiting green innovation efficiency. (3) A double-threshold effect exists with fiscal decentralization as the threshold variable. Environmental regulation showed an inverted N-shaped relationship of inhibiting, promoting, and then inhibiting green innovation efficiency. The study results provide theoretical guidance and practical reference for China to achieve the dual carbon goal.

## 1. Introduction

With the strengthening global economy, environmental pollution has become increasingly serious. In the 2030 Agenda for Sustainable Development, the United Nations set up future development goals for economic and environmental issues through which countries can continue to adjust their development models and accelerate green transformation. At present, both green innovation and green low-carbon transition have become widespread hot topics in China. At the 75th UN General Debate in September 2020, President Xi Jinping proposed that “China will adopt stronger policies and measures to strive to achieve peak carbon by 2030 and carbon neutrality by 2060”, which means that China will reach the goal of carbon neutrality in half the time compared with western countries. Thus, achieving the dual carbon targets is required for China to achieve high-quality green development and for the government to vigorously promote regional green innovation and green transformation. During the 14th Five-Year Plan period, China should promote the innovation of green technologies, such as pollution and carbon reduction, under the vision of carbon neutrality and carbon peaking to achieve green development. To achieve carbon neutrality, abandon development models that damage the ecological environment, break the old dynamics of regional development, and build a beautiful country, and the whole country should, in the context of strengthening environmental regulation, take a development path that prioritizes the ecological environment and green low-carbon innovation, insist on innovation as the first force driving development, implement the development concept of “green mountains and clear water are equal to mountains of gold and silver”, continuously optimize the ecological innovation environment, improve the regional innovation environment, and enhance the efficiency of regional green innovation.

Within this context, most researchers have explored the relationship between environmental regulation and green innovation efficiency at the micro level, such as in the construction industry [1] and heavy-polluting industries [2]. Studies exploring these aspects from the provincial macro level in China are lacking. In terms of empirical analysis methods, scholars have used Tobit regression [2] and systematic GMM [3] by introducing mediating variables without further exploring how environmental regulation affects the efficiency of green innovation from the threshold perspective. In general, many studies have been conducted on environmental regulation and green innovation efficiency. However, the extent to which environmental regulation, fiscal decentralization, and environmental protection investment can promote green innovation efficiency based on the dual carbon target strategy still needs further exploration. Given this, we aimed to answer the following questions: How does the intensity of environmental regulation affect the efficiency of green innovation in China? Does a certain threshold range exist within which the efficiency of green innovation can be improved? We aimed to provide policy insights for achieving the dual carbon goal by answering these questions.

Therefore, we focused on the core question of how to improve green innovation efficiency through environmental regulation and explored the threshold effect of environmental regulation on green innovation efficiency in 30 provinces and cities in China during 2011–2020. We followed a main logical line of model construction, empirical testing, then result analysis. We used the DEA-SBM to measure the green innovation efficiency of 30 provinces in China, introduced environmental regulation as the core explanatory variable, and environmental protection input and fiscal decentralization as the threshold variables to construct a panel threshold model. We then empirically analyzed the threshold effect of environmental regulation on green innovation efficiency. In summary, we extracted common patterns by analyzing the threshold effects; our study provides theoretical references for achieving the dual carbon goal.

## 2. Literature Review

### 2.1. Environmental Regulation and Green Innovation Efficiency

The academic research on environmental regulation and green innovation efficiency has mainly focused on the Porter hypothesis. Environmental regulation can be divided into formal and informal environmental regulation. Formal environmental regulation refers to the institutional arrangement in which the government uses policies and regulations, economic instruments, and market mechanisms to regulate the behavior of the public to protect the environment [4]. Informal environmental refers to the environmental requirements of communities, people, etc., outside the institution [5]. Environmental regulation can also be divided into three types: command, market-based, and voluntary regulation [6]. The Porter hypothesis asserts that strict environmental regulations can stimulate innovation and, therefore, may offset environmental costs and provide economic benefits to firms [7]. The academic findings fall into three broad categories. The first one shows that environmental regulation positively affects the efficiency of green innovation, which is consistent with the Porter hypothesis. Yu (2017) [8] analyzed the data from 38 cities in the Yangtze River Economic Zone and concluded that environmental regulations played a role in improving green innovation efficiency. The findings of a second study refuted the Porter hypothesis; the authors argued that environmental regulation not only does not improve green innovation performance, but also leads to a decrease in green innovation performance. Zhou (2022) [9] analyzed the empirical results and concluded that environmental regulation negatively affects green innovation efficiency. In a third study, the authors argued that the effect of environmental regulation on green innovation efficiency is uncertain. Fan (2021) [10], based on the panel data of 235 Chinese cities from 2004 to 2016, constructed a spatial error model, and a positive U-shaped relationship was found between environmental regulations and urban green innovation efficiency. As such, consensus has not been reached on the impact of environmental regulations on green innovation efficiency, and the debate on Porter’s hypothesis is ongoing.

### 2.2. Environmental Protection Inputs and Green Innovation Efficiency

Three views on the influence of environmental protection input and green innovation efficiency have been expressed. One is the opposing theory based on the traditional hypothesis, in which neoclassical economic theory states that environmental protection input will increase the economic burden and increase the cost of innovation, which is not conducive to the improvement in green innovation efficiency [11]. The second is the positive theory based on Porter’s hypothesis, in which the increase in environmental protection input helps to stimulate green innovation, form green innovation trends, accelerate the transformation of green innovation results, and improve the efficiency of green innovation [12]. For example, Tian and Zheng (2019) [13] empirically found that centralized environmental R&D investment can improve green hormone innovation efficiency in the short term. Third, based on the uncertainty hypothesis, the view is that the specific implementation process and method of environmental protection input will affect green innovation efficiency, so the relationship between the two involves uncertainty, and a threshold effect may exist. Chen (2020) [12] studied the impact of environmental inputs on enterprise productivity and concluded that the relationship is U-shaped and that only environmental inputs that cross the threshold will positively impact enterprise productivity. Scholars have studied the relationship between environmental inputs and green innovation efficiency. However, the results have varied due to differences in data selection and model settings; no unified conclusion has been reached.

### 2.3. Fiscal Decentralization and Green Innovation Efficiency

The relationship between fiscal decentralization and green innovation efficiency has received extensive academic attention. Fiscal decentralization [14] is a method of dividing the work between central and local governments, which mainly involves the central government ceding a certain degree of financial autonomy to local governments so that local governments can provide public services according to local conditions. Three main academic views exist on how fiscal decentralization affects green innovation performance. The first is promotion theory, a view that the degree of fiscal decentralization is positively related to green innovation efficiency, where the higher the degree of fiscal decentralization, the more government finance and funds support regional green innovation and the more conducive to improving regional green innovation efficiency [15]. Second is inhibitory theory; under the Chinese style of fiscal decentralization, promotion has an incentive effect on the government. Influenced by the performance view, the government may make decisions that are not conducive to the increase in green innovation efficiency [16]. The third is uncertainty theory, which argues that many factors influence the relationship between fiscal decentralization and green innovation efficiency and that spatial heterogeneity exists in the impact of fiscal decentralization on green innovation efficiency [12]. Fiscal decentralization in China is essential to China’s economic development. Although fiscal decentralization provides autonomy and convenience for local governments, it also generates a risk of self-interest preferences. Scholars have expressed their views on the relationship between fiscal decentralization and green innovation efficiency, and no unified conclusion has been formed.

By summarizing the existing literature, we found that the domestic discussion on green innovation efficiency from different perspectives has been thorough, with scholars considering the factors influencing green innovation efficiency [17,18], spatial distribution [10], and spatial and temporal characteristics [19]. The macro-level research on green innovation efficiency has been developed from the efficiency level using measurement models. In terms of measurement methods, the main focus has been on data envelopment analysis (DEA) methods and their derivatives. For example, Lv et al. (2019) [20] and Wu (2019) [21] constructed SBM-DEA models and three-stage DEA models to measure regional green innovation in China based on panel data of 30 provinces, cities, and autonomous regions in China, respectively. Fu et al. (2022) [2] established SBM models of unexpected output to measure green innovation efficiency; Xu and Zhou (2020) [22] used a three-stage Malmquist model to measure the green innovation efficiency of eight economic zones in China. In terms of efficiency level, Zhao et al. (2021) [23] analyzed 2009–2017 Chinese provincial panel data and concluded that China’s green innovation efficiency has been growing each year. Long et al. (2020) [24] and Xu et al. (2021) [25] found that Chinese regional green innovation efficiency is high in the east and low in the west.

In summary, although many empirical results on green innovation efficiency have been published, few studies have linked environmental regulation to green innovation efficiency at the provincial level. Therefore, the marginal contribution of this is a discussion of the threshold effect of environmental regulation on green innovation efficiency from dual perspectives: environmental protection input and fiscal decentralization. This study complements the literature on how environmental regulation affects green innovation efficiency at the provincial level, and we provide policy recommendations for exploring the impact of different thresholds on green innovation efficiency from the perspectives of environmental protection input and fiscal decentralization, thereby promoting sustainable development, high-quality development, and the achievement of the dual carbon goal.

## 3. Methodology and Data Sources

### 3.1. Model Construction

Considering the influence of two factors, fiscal decentralization and environmental protection input, environmental regulation may have a nonlinear relationship with green innovation efficiency in different environments. Therefore, we constructed a panel threshold model based on Hansen’s (1999) [26] panel data threshold regression model, as follows:

(1) $${\mathrm{GIE}}_{\mathrm{it}}=\mu_{i}+\beta_{1}{\mathrm{ER}}_{\mathrm{it}}I\left(Q_{\mathrm{it}}\leq \gamma_{1}\right)+\beta_{2}{\mathrm{ER}}_{\mathrm{it}}I(\gamma_{1}<Q_{\mathrm{it}}\leq \gamma_{2})+\beta_{3}{\mathrm{ER}}_{\mathrm{it}}I(Q_{\mathrm{it}}>\gamma_{2})+{\alpha Z}_{\mathrm{it}}+\varepsilon_{\mathrm{it}}$$

where i represents province; t represents year; ${\mathrm{GIE}}_{\mathrm{it}}$ represents green innovation efficiency; ${\mathrm{ER}}_{\mathrm{it}}$ represents environmental regulation; $\beta_{1}$ and $\beta_{2}$ are regression coefficients, $Q_{\mathrm{it}}$ is the threshold variable; $\gamma_{1},{\;\gamma }_{2}$ is the threshold value to be estimated; $I\left(\cdot \right)$ is the indicative function, taking 1 when a threshold value exists, and 0 otherwise; $\mu_{i}$ is the eigenvalue of observation; $Z_{\mathrm{it}}$ is the control variable; α is the control variable coefficient; $\varepsilon_{\mathrm{it}}$ is the random disturbance term. Equation (1) is a two-threshold model or a single-threshold model, if the estimation is missing in the middle.

Taking environmental protection input and fiscal decentralization as threshold variables, respectively, we explored the nonlinear relationship between environmental regulation and green innovation efficiency and constructed a double-threshold model with the following expressions:

(2) $${\mathrm{GIE}}_{\mathrm{it}}=\mu_{i}+\beta_{1}{\mathrm{IPC}}_{\mathrm{it}}I\left({\mathrm{IPC}}_{\mathrm{it}}\leq \gamma_{1}\right)+\beta_{2}{\mathrm{IPC}}_{\mathrm{it}}I(\gamma_{1}<{\mathrm{IPC}}_{\mathrm{it}}\leq \gamma_{2})+\beta_{3}{\mathrm{IPC}}_{\mathrm{it}}I({\mathrm{IPC}}_{\mathrm{it}}>\gamma_{2})+{\alpha Z}_{\mathrm{it}}+\varepsilon_{\mathrm{it}}$$

(3) $${\mathrm{GIE}}_{\mathrm{it}}=\mu_{i}+\beta_{1}{\mathrm{FD}}_{\mathrm{it}}I\left({\mathrm{FD}}_{\mathrm{it}}\leq \gamma_{1}\right)+\beta_{2}{\mathrm{FD}}_{\mathrm{it}}I\left(\gamma_{1}\leq {\mathrm{FD}}_{\mathrm{it}}\leq \gamma_{2}\right)+\beta_{3}{\mathrm{FD}}_{\mathrm{it}}I({\mathrm{FD}}_{\mathrm{it}}>\gamma_{2})+{\alpha Z}_{\mathrm{it}}+\varepsilon_{\mathrm{it}}$$

### 3.2. Variable Setting

#### 3.2.1. Explained Variable: Green Innovation Efficiency (GIE)

The DEA method is widely used in efficiency measurement, but the traditional DEA model ignores unexpected outputs and does not consider the input–output slack at the same time. The DEA-SBM unexpected output model proposed by Tone (2001) [27], which considers the inefficiency scenario from both input and output perspectives and adds slack variables to the objective function, solves the above problems and has been widely used in agriculture [28], economic development [29], and environmental resources [30,31] studies, among others. Based on this, we used a DEA-SBM model to measure the green innovation efficiency (GIE) of each province following Wang (2019) [32].

In this study, we took each province as a production decision unit, and we assumed each had M inputs, $X\in \left(x_{1},x_{2},\ldots \ldots x_{n}\right)x\in R^{m*n},(x_{i}>0)$, s outputs, $s_{1}$ desired output species ($y^{g}\in R^{s_{1}})$, and $s_{2}$ undesired output species ($y^{b}\in R^{s_{2}})$, defined as follows:

$$Y^{g}=\left(y_{1}^{g},y_{2}^{g}\ldots y_{n}^{g}\right)\in R^{s_{1}*n}$$

$$Y^{b}=\left(y_{1}^{b},y_{2}^{b}\ldots y_{n}^{b}\right)\in R^{s_{2}*n}$$

where $y_{i}^{g}>0,y_{i}^{b}>0$. Then, the DEA-SBM model is:

$$\rho^{*}=\min\rho =\min\frac{1-\frac{1}{m}\sum_{i=1}^{m}\frac{s_{i}^{-}}{x_{i0}}}{1+\frac{1}{s_{1}+s_{2}}\left\{\sum_{i=1}^{s_{1}}\frac{s_{i}^{g}}{y_{\mathrm{io}}^{g}+\sum_{i=1}^{s_{2}}\frac{s_{i}^{b}}{y_{\mathrm{io}}^{b}}}\right\}}$$

$$s.t.x_{0}=X\lambda +s^{-}$$

$$y_{0}^{g}=Y^{g}\lambda -s^{b}$$

$$y_{0}^{b}=Y^{g}\lambda +s^{b}$$

$$\lambda \geq 0,\;s^{-}\geq 0,\;s^{g}\geq 0,\;s^{b}\geq 0$$

where $s^{-}$ is the slack variable for the input, $s^{g}$ is the slack variable for the desired output, and $s^{b}$ is the slack variable for the undesired output. The objective function is strictly decreasing for the three slack variables and the function values $\rho^{*}\in \left[0,\;1\right]$.

**Figure 1:**
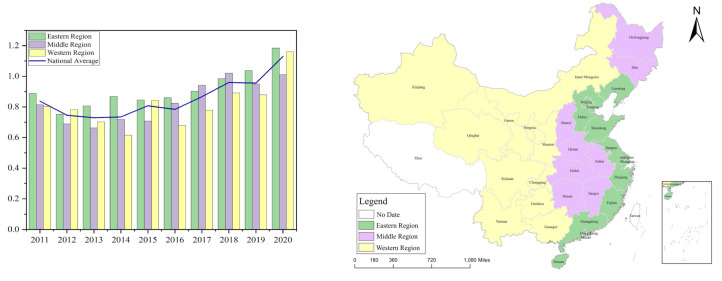
Regional green innovation efficiency comparison 2011–2020.

In this study, we defined green innovation efficiency from two perspectives: green innovation input and green innovation output, where input considers three main aspects: human, capital, and energy; output considers two main aspects: economic and environmental benefit. The definition of green innovation efficiency variables is detailed in Table 1. Moreover, the comparison of green innovation efficiency in each region is shown in Figure 1, where the eastern region includes Beijing, Tianjin, Hebei, Liaoning, Shanghai, Jiangsu, Zhejiang, Fujian, Shandong, Guangdong, and Hainan (11 provinces and cities); the central region includes Shanxi, Jilin, Heilongjiang, Anhui, Jiangxi, Henan, Hubei, and Hunan (8 provinces); the western region includes Inner Mongolia, Guangxi, Chongqing, Sichuan, Guizhou, Yunnan, Tibet, Shaanxi, Gansu, Qinghai, Ningxia, and Xinjiang (12 provinces, cities, and autonomous regions). In terms of overall efficiency, the level of green innovation efficiency in China continues to increase, the emphasis on green innovation is increasing, and the provinces are increasingly effectively implementing the green development concept. During the sample period, the level of green innovation efficiency in the country and the three main regions showed spatial differences in the national average value. The regional differences were also pronounced.

#### 3.2.2. Core Explanatory Variable: Environmental Regulation (ER)

In prior studies, scholars did not have a relatively unified standard for measuring environmental regulation. At present, environmental regulation is measured mainly from two perspectives, the first of which is the effect of environmental regulation. Liu and He (2021) [46] used single indicators, such as sulfur dioxide removal rate and industrial wastewater discharge compliance rate, to measure environmental regulation. The second is from the perspective of the intensity of environmental regulation. He et al. (2022) [47] used a relative number of indicators, the proportion of industrial pollution investment to the added value of the secondary industry, to measure environmental regulation. As we studied the influence of environmental regulation on an efficiency indicator (green innovation efficiency), we chose the share of industrial pollution investment treatment amount to the added value of secondary industry to measure this indicator.

#### 3.2.3. Core Explanatory Variable: Environmental Regulation (ER)

1. Fiscal Decentralization (FD)

The fiscal decentralization indicator reflects autonomous local finance. Researchers have measured fiscal decentralization mainly from three perspectives: fiscal revenue, fiscal freedom, and fiscal expenditure. We referred to Zhang et al. (2022) [48] to measure fiscal decentralization from the perspective of fiscal expenditure and to eliminate the influence of demographic factors, the proportion of local fiscal expenditure to the local population, and the proportion of central fiscal expenditure to the national population.

2. Environmental Protection Inputs (IPC)

Various measures are available for environmental protection input in China, such as greening coverage, the amount of high investment in urban public transportation, subsidies and investment in industry, and the amount of completed investment in environmental pollution control [49]. As our purpose of this study was to examine the influence of environmental regulation on the efficiency of green innovation, we chose the proportion of local investment in environmental pollution control to local GDP [50] to measure the environmental protection investment as an indicator.

#### 3.2.4. Control Variables

Based on prior studies and to avoid estimation bias caused by omitted variables, we included four indicators: regional economic development level, energy consumption intensity, education level, and regional openness as control variables in the analysis model. These specifically included (i) regional economic development level (GDP), expressed as regional GDP per capita; (ii) energy consumption intensity (EI), expressed as the ratio of total energy consumption to regional GDP in each province; (iii) education level (EDU), expressed as the educated level of employed persons in each province; and (iv) regional openness (OP), expressed as the ratio of the foreign direct investment amount to GDP in each province.

### 3.3. Data Sources

According to data availability, we selected the panel data of 30 provinces in China (we excluded Tibet, Hong Kong, Macao, and Taiwan because of incomplete data) from 2011 to 2020 for the study. We obtained all data from the China Environmental Yearbook, the China Population and Employment Statistical Yearbook, the China Labor Statistical Yearbook, and the China Energy Statistical Yearbook. The descriptive statistics for each variable are shown in Table 2. To eliminate heteroskedasticity, we log-transformed some of the variables. As shown in Table 3, the correlation among the variables was significant, and the maximum VIF value was 3.65, which is much less than 10, indicating no multicollinearity.

## 4. Empirical Analysis

### 4.1. Stability Test

To avoid pseudo-regression and increase the smoothness of the panel data, we conducted the unit root test on the panel data of 30 provinces. In this study, we chose the Levin–Lin–Chu test for the unit root test. Table 4 shows the results of the smoothness test of the variables, demonstrating that all variables were significant at the 1% statistical level, so we found no unit root. That is, all variables were smooth variables.

### 4.2. Threshold Effect Test

According to Models (2) and (3), we used STATA16 to explore the effect of environmental regulation on green innovation efficiency, with environmental protection input (IPC) and fiscal decentralization (FD) as the threshold variables, respectively. First, we estimated the threshold values and tested them for significance. As shown in Table 5, the *p*-value of the single-threshold test for Model (2) with environmental protection input as the threshold variable was 0.000 (0.000 ≤ 0.000), which was significant at the 1% statistical level, indicating the existence of a single-threshold effect. Second, we proceeded with the double-threshold test. The results showed that the *p*-value of the double-threshold test was 0.000 (0.000 ≤ 0.000), which was significant at the 1% statistical level, indicating a double-threshold effect. Similarly, the *p*-value of the double threshold test for Model (3) with fiscal decentralization as the threshold variable was 0.000 (0.000 ≤ 0.000), indicating a double-threshold effect in Model (3). Furthermore, Table 6 exhibits the threshold point estimates and confidence intervals (under the 95% level). The narrow confidence intervals of the four thresholds further proved the significance of the threshold effect, indicating a nonlinear relationship between environmental regulations and green innovation efficiency in each province.

The estimation results of the threshold values in Table 6 show that in Model (2), with environmental protection input as the threshold variable in the study sample, the three eastern coastal regions, Shanghai, Tianjin, and Jilin, had lower environmental protection input than the first threshold during 2017–2019. Hainan province had lower environmental protection input than the first threshold during 2014–2016, which indicated that the local environmental protection in these regions during this period investment was weak. Qinghai and Guangdong were below the second threshold in 2015, whereas environmental protection investment in the eastern regions of Beijing, Jiangsu, Zhejiang, and Shandong was consistently higher than the second threshold, which suggested that these regions focused on spending on environmental pollution control and environmental protection investment. For Model (3), when using fiscal decentralization as the threshold variable, Qinghai has exceeded the second threshold in 2011–2015; however, afterward, Qinghai was below the first threshold, indicating that the degree of local fiscal decentralization showed a trend of first increasing and then decreasing. During 2011–2020, the eastern coastal regions, such as Jilin, Heilongjiang, Liaoning, Tianjin, and Shandong, and some central and western regions such as Sichuan, Chongqing, and Shanxi, were lower than the first threshold. Eastern regions such as Beijing and Shanghai broke the first threshold during 2011–2015 and the second threshold during 2016–2020, indicating that the fiscal decentralization capacity of these regions was strong and continuously improving and optimizing.

The regression results of the panel threshold model are shown in Table 7, which indicated that environmental regulation had a nonlinear relationship with green innovation efficiency. As the threshold variables, environmental protection input and fiscal decentralization, changed from weak to strong, environmental regulation showed an inverse N-shaped relationship of first inhibiting, then promoting, and finally inhibiting green innovation efficiency. Table 7 reports the effect of environmental regulation on green innovation efficiency for different threshold intervals for Models (2) and (3), showing that environmental regulation inhibited green innovation efficiency in provinces where environmental protection input was below the first threshold value of 0.0234, but this result did not pass the significance test. Environmental regulation promoted green innovation efficiency when environmental protection input exceeded the first threshold value of 0.0234 and promoted green innovation efficiency when the estimated coefficient decreased from −13.932 to −29.262, environmental protection input exceeded the second threshold value of 0.0250, and environmental regulation inhibited green innovation efficiency. The inhibitory effect of environmental regulation on regional green innovation was exacerbated at this point when environmental protection inputs are below the first threshold. For provinces with a fiscal decentralization degree below the first threshold of 2.2749, where environmental regulation inhibited the increase in green innovation efficiency when the first threshold value of 2.2749 was breached, environmental regulation played a role in facilitating green innovation efficiency. When the second threshold value of 2.4352 was breached, environmental regulation played an inhibiting, not significant, role in green innovation efficiency.

### 4.3. Robustness Test

To further prove the preceding conclusions and consider the possible time-lag effects of the threshold variables, environmental protection input and fiscal decentralization, on green innovation efficiency, we adopted an extended time window to conduct robustness tests by treating the threshold variables (environmental protection input and fiscal decentralization) with one and two lags, respectively. The robustness tests involved the following four models:

(4) $${\mathrm{GIE}}_{\mathrm{it}}=\mu_{i}+\beta_{1}L.{\mathrm{IPC}}_{\mathrm{it}}I\left(L.{\mathrm{IPC}}_{\mathrm{it}}\leq \gamma_{1}\right)+\beta_{2}L.{\mathrm{IPC}}_{\mathrm{it}}I(\gamma_{1}<L.{\mathrm{IPC}}_{\mathrm{it}}\leq \gamma_{2})+\beta_{3}L.{\mathrm{IPC}}_{\mathrm{it}}I(L.{\mathrm{IPC}}_{\mathrm{it}}>\gamma_{2})+{\alpha Z}_{\mathrm{it}}+\varepsilon_{\mathrm{it}}$$

(5) $${\mathrm{GIE}}_{\mathrm{it}}=\mu_{i}+\beta_{1}L2.{\mathrm{IPC}}_{\mathrm{it}}I\left(L2.{\mathrm{IPC}}_{\mathrm{it}}\leq \gamma_{1}\right)+\beta_{2}L2.{\mathrm{IPC}}_{\mathrm{it}}I(\gamma_{1}<L2.{\mathrm{IPC}}_{\mathrm{it}}\leq \gamma_{2})+\beta_{3}L2.{\mathrm{IPC}}_{\mathrm{it}}I(L2.{\mathrm{IPC}}_{\mathrm{it}}>\gamma_{2})+{\alpha Z}_{\mathrm{it}}+\varepsilon_{\mathrm{it}}$$

(6) $${\mathrm{GIE}}_{\mathrm{it}}=\mu_{i}+\beta_{1}L.{\mathrm{FD}}_{\mathrm{it}}I\left(L.{\mathrm{FD}}_{\mathrm{it}}\leq \gamma_{1}\right)+\beta_{2}L.{\mathrm{FD}}_{\mathrm{it}}I\left(\gamma_{1}\leq L.{\mathrm{FD}}_{\mathrm{it}}\leq \gamma_{2}\right)+\beta_{3}L.{\mathrm{FD}}_{\mathrm{it}}I(L.{\mathrm{FD}}_{\mathrm{it}}>\gamma_{2})+{\alpha Z}_{\mathrm{it}}+\varepsilon_{\mathrm{it}}$$

(7) $${\mathrm{GIE}}_{\mathrm{it}}=\mu_{i}+\beta_{1}L2.{\mathrm{FD}}_{\mathrm{it}}I\left(L2.{\mathrm{FD}}_{\mathrm{it}}\leq \gamma_{1}\right)+\beta_{2}L2.{\mathrm{FD}}_{\mathrm{it}}I\left(\gamma_{1}\leq L2.{\mathrm{FD}}_{\mathrm{it}}\leq \gamma_{2}\right)+\beta_{3}L2.{\mathrm{FD}}_{\mathrm{it}}I(L2.{\mathrm{FD}}_{\mathrm{it}}>\gamma_{2})+{\alpha Z}_{\mathrm{it}}+\varepsilon_{\mathrm{it}}$$

Models (4) and (5) are based on the model with environmental protection input as the threshold variable, replacing the threshold variable with one period lagged and two periods lagged, respectively, where $L.{\mathrm{IPC}}_{\mathrm{it}}$ represents the environmental protection input with one period lagged and $L2.{\mathrm{IPC}}_{\mathrm{it}}$ represents the environmental protection input with two periods lagged. Similarly, Models (6) and (7) are based on the model with fiscal decentralization as the threshold variable, and replacing the threshold variables with one period lagged and two periods lagged, respectively. $L.{\mathrm{FD}}_{\mathrm{it}}$ represents fiscal decentralization with one period lagged, $L2.{\mathrm{FD}}_{\mathrm{it}}$ represents fiscal decentralization with two periods lagged, and $Z_{\mathrm{it}}$ is the control variable. The results of the threshold effect test for the lagged variables are shown in Table 8.

According to the test, in Models (4) to (7), all of which have double-threshold effects, as the intensity of the lagged threshold variables, environmental protection input and fiscal decentralization, changed from weak to strong, the effect of environmental regulation on green innovation efficiency presented an inverse N-shaped relationship of first inhibiting, then promoting, and finally inhibiting. This finding is consistent with the conclusion drawn in a previous study; that is, the threshold regression model in this study is robust (Table 9).

## 5. Discussion

First, during 2011–2020, the average green innovation efficiency in the central and western regions irregularly fluctuated compared with the national average; in the eastern region, the average green innovation efficiency was higher than the national average. Thus, we found that development of regional green innovation was uneven, being strong in the east and weak in the west. This is roughly the same as the findings of Wang (2019) [32], which indicates that green innovation efficiency is closely related to regional development, and the eastern region is leading in terms of economic foundation, institutional environment or scientific and technological R&D, and has obvious advantages in green innovation development. The central and western regions are mainly characterized by high-pollution and high-energy-consumption industries, and their green innovation foundation is weaker, so their green innovation development is in the initial stage. This finding is similar to that of Zhang (2022) [19], who argued that the eastern region in China is consistently leading in innovation, whereas the central and western regions differ in terms of resource endowment, green innovation investment, and geographic location, so they face different problems and have different advantages when attempting to increase the efficiency of green innovation.

Second, using environmental protection input as the threshold variable, we found that the effect on green innovation efficiency was significant at the 1% statistical level, which supported a double-threshold effect. The effect of environmental regulation on green innovation efficiency is an inverted N-shape: first inhibiting, then promoting, and finally inhibiting. In provinces with environmental protection investment below the first threshold, for example, Shanghai, Tianjin, and Jilin, and other provinces and cities that accelerated industrial transformation and upgrading during 2017–2019, environmental regulation played an inhibitory role in green innovation efficiency but did not pass the significance test, probably because regions below the first threshold, whose governments did not focus on the treatment of environmental pollution during 2017–2019, invested less in environmental protection. The relationship between the intensity of environmental regulation, green innovation efficiency, and environmental protection investment was not strong in this period. Regions between the two thresholds, such as Guangdong and Qinghai, responded to the call of China’s 12th and 13th Five-Year Plans during the sample period to strengthen the cooperation among resources, finance, trade, and tourism to maximize the benefits and increase the investment in environmental protection, which, in turn, stimulated local green innovation. In this case, environmental regulation played a main role in promoting the efficiency of green innovation. In regions where environmental protection investments exceeded the second threshold, such as Beijing, Jiangsu, Zhejiang, Fujian, and other economically developed eastern regions, local governments had sufficient funds to invest in environmental protection, but environmental regulations inhibited green innovation efficiency at this time, probably because as the demand for investment in environmental management increased, funding support for green innovation technologies was neglected or reduced due to the uneven distribution of funds. Our conclusion regarding this inverted N-shaped relationship is identical to that found by Shen (2022) [3]. However, the mechanism of the direct impact of environmental protection inputs on the efficiency of green innovation needs to be further analyzed.

Third, when fiscal decentralization was used as the threshold variable, it had a significant effect on green innovation efficiency at the statistical levels of 10% and 1%. This indicated a double-threshold effect, where the effect of environmental regulation on green innovation efficiency showed an inverted N-shaped relationship of first inhibiting, then promoting, and finally inhibiting. For the green innovation efficiency for provinces with fiscal decentralization below the first threshold, such as Heilongjiang, Jilin, and Liaoning, environmental regulation had a significant inhibitory effect on green innovation efficiency, probably because of the persistent low regional GDP due to overcapacity, and the special decentralization system of “responsibility at the grassroots and purse at the top”, which is characteristic of Chinese fiscal decentralization. This results in a small degree of regional fiscal decentralization, and the local governments do not have enough funds to attract dynamic green innovation enterprises, which inhibits the development of green innovation. This is broadly similar to the conclusion of Dong (2022) [51], who argued that economic catch-up among local governments substantially reinforces the inhibitory effect of fiscal decentralization on green innovation additionally, and that government investment preferences, foreign technology selection paths, and regional heavy-polluting industries are the main influencing factors. For Beijing, Shanghai, and Guangdong, the three first-tier development regions in China, which were between the two thresholds of fiscal decentralization during 2011–2015, the degree of fiscal decentralization was higher, and environmental regulation played a notable role in promoting green innovation efficiency. This is similar to the findings of Li (2018) [52], who argued that fiscal decentralization promotes the financial autonomy of local governments, and local governments have the information advantage of being well-informed about the local situation, which ensures that local governments can play a leading role in the innovation activities in the region and increase the efficiency of regional innovation. However, during 2016–2020, in Beijing, Shanghai, and Guangdong provinces, fiscal decentralization broke through the second threshold. At this time, environmental regulation played a large role in promoting green innovation efficiency. Environmental regulation played a suppressive, but nonsignificant, role in green innovation efficiency; the possible reason for this finding is that these three regions have more local autonomy in the process of shifting from the new economic normal of the 12th Five-Year Plan to the stage of high-quality economic development in the 13th Five-Year Plan, but the relationship between the degree of fiscal decentralization, environmental regulation, and green innovation efficiency was not strong at this time.

## 6. Conclusions, Policy Implications, and Limitations

### 6.1. Conclusions

We measured the green innovation efficiency in China through a DEA-SBM model, and we empirically analyzed the results based on the panel data of 30 provinces in China from 2011 to 2020 to explore the threshold effect of environmental protection input and fiscal decentralization in environmental regulation, affecting green innovation efficiency. The results are as follows:

First, from 2011 to 2020, the green innovation efficiency of Chinese provinces was trending upward, and the level of green innovation efficiency in each region was spatially heterogeneous, being strong in the east and weak in the middle and west.

Second, we found a nonlinear relationship between environmental regulation and green innovation efficiency for the 30 provinces and cities in China during 2011–2020. We identified a double-threshold effect with environmental inputs as the threshold variable, as well as an inverted N-shaped relationship between environmental regulations on green innovation efficiency.

Third, we found a double-threshold effect with fiscal decentralization as the threshold variable, with an inverted N-shaped relationship between environmental regulations and green innovation efficiency.

### 6.2. Policy Implications

Based on the results of the empirical analysis, we developed the following policy implications from the perspective of improving ecological and environmental protection and enhancing the efficiency of regional green innovation:

First, the following are required: improving local ecological and environmental protection measures, completing the local budget management system, and establishing a system of synergistic development between them. We should improve the local government’s budgetary expenditure management system and increase the scale and proportion of special funds for environmental protection according to local resources and advantages, as well as improve the reasonable supervision system. At present, the Chinese budget monitoring system is not sufficiently transparent and binding, so local governments may divert funds meant for ecological and environmental management to other projects to improve their performance. Additionally, we should establish an ecological and environmental management system with synergy among economic and ecological development, so that each local government can ensure the implementation of effective environmental protection measures according to its characteristics and stimulate the enthusiasm to engage in ecological and environmental management. While promoting Chinese economic development from the stage of high-speed growth to the stage of high-quality development, we should create appropriate defenses of the environmental ecosystem.

Second, an incentive policy for differentiated green innovation should be formulated to guide the synergistic development of regional scientific and technological innovation and environmental protection, as well as to strengthen regional innovation capacity building and talent training. During the sample period in this study, the eastern region of China had the highest green innovation efficiency; thus, the eastern region should use its regional advantages to encourage the increase in independent innovation capacity and advocate for the environment to promote the green innovation efficiency while stabilizing the high-quality economic development. The central and western regions in China had lower green innovation efficiency, based on which they should use their rich resources and technological opportunities to increase the regional environmental protection efforts. Based on the economic development trend, they should break the original fixed pattern and expand incentive policies, such as by increasing policy preferences, introducing scientific and technological talent, orienting and training scientific and technological talent, and attracting high-tech environmental management technologies from other developed countries to provide personnel and policy guarantees for the development of regional green innovation projects and fully use the technology spillover effect to promote the achievement of the dual carbon goals.

Third, the Chinese fiscal decentralization system should be improved, the construction of a modern fiscal system should be accelerated, and the reform of the fiscal and taxation system should be deepened. Additionally, the division of fiscal affairs and responsibilities between the central and local governments should be completed. Either too low or too high a degree of fiscal decentralization will inhibit green innovation efficiency. Therefore, the central government’s commitment to providing basic public services should be moderately increased, the central government’s direct involvement in grassroots finance should be reduced, and the local government’s fiscal freedom should be controlled within a reasonable range. Moreover, the local government should completely fulfill its responsibility for affairs within the central government’s authorization, take advantage of its knowledge of local resources and development, and manage their affairs according to local conditions to better focus on the goal of achieving green and low-carbon goals and promoting the efficiency of green innovation.

Fourth, we should control the strength of environmental regulations and add fiscal and taxation policies and environmental protection investments to promote the efficiency of regional green innovation, ensure the sustainability of environmental regulations, and avoid too strict and too quick enforcement. Therefore, we must implement appropriate environmental regulation policies for regions with different economic development conditions; effectively increase the investment in human, capital, and energy; increase the policy support for the research and development of low-carbon and green innovation technologies; and increase the application and promotion of these technologies to help promote green development strategies, along with high-quality economic development.

### 6.3. Limitations

Due to the constraints of data availability and study length, this study has certain shortcomings that can be further explored in the future, mainly in the following aspects: First, the relationship between the intensity of environmental protection inputs, the intensity of fiscal decentralization, and the green innovation efficiency, such as regional and spatial distribution patterns, still needs to be further explored. Second, environmental regulation has mainly been measured from two aspects: intensity and effect. In this study, we only considered one aspect: the intensity of environmental regulation. In future studies, we can measure environmental regulation from different perspectives to further improve our understanding of the mechanism through which environmental regulation impacts green innovation efficiency.

## Figures and Tables

**Table 1:** Definition of green innovation efficiency variables.

	Variables	Variable Definition	Abbreviation	References
Input	Human Input	R&D staff full-time equivalent	SFTE	Zhang and Zhu [33], Li et al. [34]
		Internal expenditure on R&D expenses	IE	Zhang and Zhu [33], Li et al. [34]
		New product development expenses	NPDE	Luo and Zhang [35]
	Capital Input	Technology introduction and renovation expenses	TIRE	Cao and Su [36]
		Environmental pollution control expenses	EPCE	Fu et al. [37]
	Energy Input	Total industrial energy consumption	TIEC	Han et al. [38], Wu et al. [39]
		Number of valid invention patents	VIP	Qian et al. [40], Chai et al. [41]
Output	Economic Benefits	New product sales revenue	NPSR	Cao and Yu [42]
		Industrial value added	IVA	Liu et al. [43]
	Environmental Benefits	Industrial wastewater discharge	IWD	Cao et al. [44], Liu et al. [43]
		Industrial waste gas emissions	IWGS	Liu et al. [43]
		Industrial solid waste emissions	IWWE	Wang et al. [45], Liu et al. [43]

**Table 2:** Descriptive statistics of study variables.

Variable	Abbreviation	Obs	Mean	SD	Min	Max
Green Innovation Efficiency	GIE	300	0.859	0.360	0.120	4.223
Environmental Regulation	ER	300	0.003	0.003	0.000	0.025
Fiscal Decentralization	FD	300	1.182	0.477	0.634	2.574
Environmental Protection Inputs	IPC	300	0.762	2.505	0.001	28.736
Economic Development Level	GDP	300	10.790	0.442	9.682	12.013
Education Level	EDU	300	10.114	1.061	8.085	14.185
Energy Consumption Intensity	EI	300	−3.628	2.971	−10.690	10.860
Regional Openness	OP	300	0.019	0.015	0.000	0.080

**Table 3:** Correlation coefficients and multiple covariance tests.

Variable	GIE	ER	FD	IPC	GDP	EDU	EI	OP
GIE	-							
ER	−0.212 ***	1.380						
FD	0.164 ***	0.203 ***	1.930					
IPC	−0.247 ***	0.245 ***	−0.056	1.510				
GDP	0.203 ***	−0.267 ***	0.390 ***	0.013	2.930			
EDU	0.099 *	−0.173 ***	0.555 ***	0.045	0.799 ***	3.650		
EI	0.147 **	0.095	0.104 *	0.012	−0.060	−0.046	1.120	
OP	−0.034	−0.197 ***	0.209 ***	0.125 **	0.353 ***	0.372 ***	−0.234 ***	1.380

Note: The diagonal is the variance inflation factor. Its maximum value is 3.65 (3.65 < 10), and no multicollinearity exists. * *p* < 0.1, ** *p* < 0.05, *** *p* < 0.01.

**Table 4:** Panel unit root test results.

Variable	LLC	Smooth Variable?
GIE	−15.553 ***	Yes
ER	−26.401 ***	Yes
FD	−16.762 ***	Yes
IPC	−14.077 ***	Yes
GDP	−13.830 ***	Yes
EDU	−12.728 ***	Yes
EI	−15.846 ***	Yes
OP	−12.068 ***	Yes

Note: *** indicate significant at 1% confidence level.

**Table 5:** Existence tests of threshold effects for Models (2) and (3).

		Single-Threshold Test	Double-Threshold Test
Model (2)	F-value	13.930	41.520
	Bootstrap *p*-value	0.000	0.000
	Crit10	10.260	11.581
	Crit5	12.651	14.122
	Crit1	17.597	18.302
	BS	500	500
	Trim	0.010	0.010
	Grid samples	1000	1000
Model (3)	F-value	12.530	24.660
	Bootstrap *p*-value	0.082	0.000
	Crit10	13.989	14.869
	Crit5	17.263	19.306
	Crit1	24.830	25.711
	BS	500	500
	Trim	0.010	0.010
	Grid samples	1000	1000

**Table 6:** Estimation results of threshold values for Models (2) and (3).

		Threshold Value	95% Confidence Interval
Model (2)	Th-1	0.023	[0.022, 0.026]
	Th-21	0.023	[0.022, 0.024]
	Th-22	0.025	[0.024, 0.026]
Model (3)	Th-1	2.275	[2.249, 2.416]
	Th-21	2.413	[2.284, 2.416]
	Th-22	2.435	[2.318, 2.456]

**Table 7:** Estimation results of panel threshold model.

Variable	Model (2)	Model (3)
GDP	0.392 *** (0.099)	0.396 *** (0.111)
EDU	−0.049 (0.063)	−0.519 * (0.367)
EI	−0.020 *** (0.006)	−0.015 *** (0.007)
OP	0.622 (1.783)	0.705 * (1.987)
ER × I (Q ≤ γ_1_)	−13.932 (18.226)	−31.999 *** (8.359)
ER × I ($\gamma_{1}$ < Q ≤ $\gamma_{2}$)	435.670 *** (36.216)	299.631 *** (36.360)
ER × I (Q > $\gamma_{2})$	−29.262 *** (7.272)	−11.015(21.062)
Cons	−2.742 *** (0.735)	−2.775 *** (0.819)
Obs.	300	300

Note: * *p* < 0.1, and *** *p* < 0.01. Standard errors are in parentheses.

**Table 8:** Existence test for lagged variable threshold effects.

Lagged Variable	Threshold	F-Value	*p*-Value	Critical Value			Threshold Value	95% Confidence Interval
				10%	5%	1%		
L.IPC	Single	15.690 ***	0.000	10.812	15.095	20.358	0.021	[0.021, 0.021]
	Double	26.290 ***	0.000	11.219	14.509	24.665	0.022	[0.022, 0.023]
L2.IPC	Single	10.280 ***	0.036	9.544	13.062	17.060	0.029	[0.028, 0.030]
	Double	16.450 ***	0.000	16.414	23.966	38.515	0.031	[0.029, 0.032]
L.FD	Single	8.480 ***	0.020	8.888	11.071	24.323	0.211	[0.020, 0.022]
	Double	11.170 ***	0.080	10.217	14.124	21.273	0.244	[0.023, 0.025]
L2.FD	Single	7.590 ***	0.030	9.884	12.234	14.273	0.028	[0.027, 0.032]
	Double	16.220 ***	0.000	11.212	15.073	29.852	0.032	[0.029, 0.032]

Note: *** significant at 1% confidence level.

**Table 9:** Robustness test results.

Variable	Model (4)	Model (5)	Model (6)	Model (7)
GDP	0.440 *** (0.125)	0.799 *** (0.163)	0.470 *** (0.145)	0.795 *** (0.163)
EDU	−0.010 (0.073)	−0.173 ** (0.087)	−0.056 (0.084)	−0.173 ** (0.367)
EI	0.015 ** (0.007)	0.021 *** (0.008)	0.019 ** (2.447)	0.022 *** (0.008)
OP	1.816 (2.108)	1.572 (5.450)	1.366 (0.006)	1.730 (1.987)
ER × I (Q ≤ γ_1_)	−36.209 (38.850)	−47.160 *** (19.517)	−64.429 (45.163)	−47.477 *** (19.558)
ER × I (γ_1_ < Q ≤ γ_2_)	336.160 *** (35.326)	192.726 *** (32.109)	84.247 *** (24.614)	188.954 *** (32.009)
ER × I (Q > γ_2_)	−23.505 *** (8.121)	−15.578 (9.975)	−29.048 *** (9.548)	−15.072 (10.001)
Cons	−3.726 *** (0.988)	−5.954 *** (1.377)	−3.544 *** (1.150)	−5.913 *** (1.380)
Obs.	270	240	270	240

Note: ** *p* < 0.05, and *** *p* < 0.01. Standard errors are in parentheses.

## Data Availability

Not applicable.
